# Preparation, Characterization, and Evaluation of Breviscapine Nanosuspension and Its Freeze-Dried Powder

**DOI:** 10.3390/pharmaceutics14050923

**Published:** 2022-04-24

**Authors:** Ting Zhang, Xixi Li, Juewen Xu, Jingbao Shao, Meihong Ding, Senlin Shi

**Affiliations:** School of Pharmaceutical Sciences, Zhejiang Chinese Medical University, Hangzhou 310053, China; zhangting55@zcmu.edu.cn (T.Z.); 202011113511011@zcmu.edu.cn (X.L.); 202011113511019@zcmu.edu.cn (J.X.); 201711113511362@zcmu.edu.cn (J.S.); 20151057@zcmu.edu.cn (M.D.)

**Keywords:** breviscapine, nanosuspension, Box–Behnken design

## Abstract

As a biopharmaceutics classification system (BCS) class IV drug, breviscapine (Bre) has low solubility in water, poor chemical stability, a short biological half-life and rapid removal from plasma. This paper prepared a Bre nanosuspension (Bre-NS) by an ultrasound-assisted anti-solvent precipitation method. Characterization of Bre-NS was studied using a Box–Behnken design concerning drug concentration in DMSO, an anti-solvent-to-solvent ratio, and sonication time. Under the optimized conditions of 170 mg/mL for the drug concentration, a 1:60 solvent-to-anti-solvent ratio, and a 9 min sonication time, the particle size of Bre-NS was 303.7 ± 7.3 nm, the polydispersity index was 0.178 ± 0.015, and the zeta potential was −31.10 ± 0.26 mV. Combined with the results from differential scanning calorimetry (DSC), powder X-ray diffraction (PXRD), and Fourier transform-infrared spectroscopy (FT-IR), the findings indicated that the crystal form and chemical structure of Bre-NS did not change during the entire process. The optimized formulation displayed good stability, increased solubility, and better in vitro release. Therefore, the results of this study can be a reference for the delivery system design of insoluble active components and effective parts in traditional Chinese medicine.

## 1. Introduction

Despite the progress of modern medicine, cardiovascular and cerebrovascular diseases (CVDs) are by far the most frequent causes of death worldwide [1]. CVDs are a serious threat to the life and health of modern people [2]. At the same time, patients, families, and society are under great pressure because of the long course of CVDs, the long treatment cycle, and high medical costs. Ischemic diseases such as myocardial infarction and cerebral infarction are common CVDs, and drugs have always been one of the most important means to prevent and control ischemic diseases [3].

Breviscapine (Bre) is a flavonoid mixture extracted from the Chinese herb Erigeron breviscapus (Vant.) Hand-Mazz [4]. Its activities are mainly attributed to the presence of a large amount (>85%) of scutellarin (scutellarein-7-O-d-glucuronide) [5]. Bre can increase blood flow, improve microcirculation, dilate blood vessels, decrease blood viscosity, promote fibrinolysis, inhibit platelet aggregation, inhibit thrombosis formation, and more [6,7,8]. Bre has been widely used in the clinical treatment of CVD [9]. However, CVD is a chronic disease that requires good drug bioavailability and constant treatment levels. Previous studies have reported that, as a biopharmaceutics classification system (BCS) class IV drug, Bre had low solubility in water, poor chemical stability, and a short biological half-life, and is rapidly removed from plasma [10,11]. Domestic and foreign researchers have proposed different countermeasures, such as the preparation of Bre into lipid emulsions or solid lipid nanoparticles to improve the solubility and bioavailability of Bre [12,13]. However, these methods were complex, the stability of the drugs was poor, the drug loading was low, and the solubility and bioavailability of the drugs were difficult to improve. Therefore, a new delivery system is urgently needed to improve the solubility, dissolution rate, safety, and drug delivery capacity of Bre.

A nanosuspension (NS) is a submicron colloidal dispersion system formed by drug nanoparticles and minimal stabilizers [14]. It can be applied to any pharmaceutical formulations, including oral, parenteral, dermal, pulmonary, ocular, and nasal administrations [15,16,17,18]. NS systems have many advantages over other drug delivery systems: they not only effectively improve the solubility and bioavailability of almost all low-solubility drugs but also have a large drug-loading capacity, a simple preparation process, and high dispersion, making them suitable for the preparation of BCS class II and IV compounds. Therefore, NS is a suitable pharmaceutical dosage form for Bre [19,20,21,22].

The preparation technology of NS can be divided into top-down and bottom-up methods [17]. In the top-down approach, two methods have been commonly used: wet stirred media milling (WSMM) and high-pressure homogenization [23]. WSMM is the most widely used and preferred method for preparing drug nanosuspensions because the top-down approach can efficiently produce small particles and does not require organic solvents, making them more viable in industrial production. However, these technologies involve high-energy inputs that generate heat, making it difficult to handle heat-sensitive materials. In addition, large amounts of energy can produce amorphous particles, causing crystal form change [24]. The bottom-up methods use dissolved drug molecules to precipitate nanoparticles in the form of evaporative solvents, supercritical fluids, anti-solvent precipitates, and chemical precipitates [25,26]. Anti-solvent precipitation (AP) is the most commonly used, effective, low-cost bottom-up technology to prepare an NS [27]. AP is the primary method of producing drug nanoparticles by mixing an organic solvent containing water-insoluble drugs with an aqueous solution [28]. It has the advantages of requiring simple technology, low energy consumption, and low equipment cost [29]. Studies have shown that, compared with other methods, the AP method can fine-tune the product’s characteristics, such as morphology and crystallinity, and improve the apparent solubility of the drug [30]. However, this method typically results in rapid particle growth, wide particle size distribution, difficult removal of organic solvents, and stability issues [31]. Sonication has been applied to AP technology. It can enhance particle size reduction and control nucleation and crystallization, promote the nucleation process and the preparation of small particles, and offer a narrow particle size distribution [28,32,33]. In this study, Bre nanosuspensions (Bre-NSs) are prepared by ultrasound-aided anti-solvent precipitation. The Box–Behnken design (BBD) is a common method of prescription process optimization, and it has obvious advantages in the process of prescription optimization. It can consider the linear and square effects of the changes in the values of the independent variables and the influence of their linear interactions [34,35]. The NS is a thermodynamically unstable system. To improve the physical stability of Bre-NS, the freeze-drying method is used to cure Bre-NS.

In this study, using the theory of the NS drug delivery system, Bre-NS was prepared by an ultrasound-assisted AP method with a small amount of surfactant/polymer as a stabilizer. BBD response surface methodology was used to optimize Bre-NS. The characterization and evaluation of Bre-NS were investigated. 

## 2. Materials and Methods

### 2.1. Materials

Bre (purity >98%) was purchased from Xi’an Tongze Biotechnology Co., Ltd. (Xi’an, China). Scutellarin (purity, 98.10%) was purchased from Chengdu Manster Biotechnology Co., Ltd. (Chengdu, China). Dimethyl sulfoxide (DMSO), N, N-dimethylformamide, and acetic acid were obtained from Guangdong Guanghua Technology Co., Ltd. (Guangdong, China). PEG4000, PEG400, and D-alpha tocopheryl polyethylene glycol succinate were obtained from Shanghai Aladdin Biochemical Technology Co., Ltd. (Shanghai, China). Poloxamer188 (Pluronic F68, F BASF, Ludwigshafen, Germany) and yolk lecithin were purchased from Shanghai Yuanye Biotechnology Co., Ltd. (Shanghai, China). Pluronic F127 was purchased from Shanghai Civic Chemical Co., Ltd. (Shanghai, China). Soybean phosphatide was supplied by Novi Bomei Pharmaceutical Technology Co., Ltd. (Beijing, China). Tween 80 and Tween 20 were purchased from Shanghai Bioengineering Co., Ltd. (Shanghai, China). Commercially available Bre injection was purchased from Kunming Longjin Pharmaceutical Co., Ltd. (Kunming, China). All other reagents were either of analytical or chromatographic grade. Double-distilled water was used throughout the experiment.

### 2.2. Cell Lines and Animals

Pheochromocytoma cell line 12 (PC12) were provided by the Cell Bank of the Chinese Academy of Sciences (Shanghai, China). The cell line was cultured in Dulbecco’s modified Eagle’s medium (DMEM) supplemented with 10% fetal bovine serum (FBS), 100 U/mL of penicillin, and 100 µg/mL of streptomycin.

Male Sprague-Dawley mice (aged 6–7 weeks) were provided by the Laboratory Animal Center of Zhejiang Chinese Medical University. All the animal experiments were performed in compliance with the Animal Laboratory Ethical Committee of the Zhejiang Chinese Medical University. The ethics approval number for this study was IACUC-20220406-17.

### 2.3. Preparation of Bre Nanosuspension

NS was prepared using an ultrasonic-aided AP method [36]. Bre was soluble in DMSO as an organic phase (solvent). The soybean phospholipid is dissolved separately in ultrapure water as an aqueous phase (anti-solvent). Both were filtered by a 0.22 μm microporous membrane. During ice bath ultrasound, the organic phase was rapidly injected into the water phase, precooled to 4 °C. After the ultrasound, the sample was immediately centrifuged, and the supernatant containing DMSO was removed. The supernatant was centrifuged twice with the same amount of ultrapure water, shaken well, and poured into a small beaker to obtain Bre-NS.

### 2.4. Measurement of the Particle Size, Polydispersity Index and Zeta Potential

The particle size, polydispersity index (PDI), and zeta potential of each formulation were detected by dynamic light scattering (DLS; Zetasizer Nano ZS90, Malvern Instruments, Worcestershire, UK). All samples were diluted to an appropriate concentration with ultrapure water and tested in triplicate. All samples were measured at 25 °C with a detector angle of 90°, a wavelength of 633 nm, a refractive index of 1.590, and an imaginary particle refractive index = 0.010.

### 2.5. Investigation of Formulation Parameters

#### 2.5.1. Choice of Drug Concentration in a Good Solvent

Nanocrystals were prepared using an ultrasonic-aided anti-solvent precipitation method. Bre was dissolved in DMSO to obtain solutions with drug concentrations of 50, 100, 200, 300, and 400 mg/mL, which represented the organic phase (solvent). In addition, soybean lecithin was dissolved in ultrapure water to obtain a stabilizer solution with a concentration of 0.5% (*w*/*v*), representing the aqueous phase (anti-solvent). Both phases were filtered by a 0.22 μm microporous membrane. The ratio of solvent to anti-solvent (*v*/*v*) was 1:40. The sonication power was set to 200 W (20–25 kHz, Ningbo Scientz Biotechnology Co. Ltd., Ningbo, China), and the probe was submerged midway within the liquid level. During an ice water bath ultrasound, the drug solution was rapidly injected into 20 mL of stabilizer solution that was precooled to 4 °C, and the sonication time was performed for 5 min as continuous bursts of 5 s each with 5 s intervals.

Then, the samples were centrifuged twice (12,000 rpm, 10 min) to remove DMSO. Bre-NS was obtained. All tests have been conducted in triplicate.

#### 2.5.2. Anti-Solvent-to-Solvent Ratio

The drug concentration of Bre was 100 mg/mL as the organic phase (solvent). Soybean lecithin was dissolved in ultrapure water to obtain a stabilizer solution with a concentration of 0.5% (*w*/*v*) as the aqueous phase (anti-solvent). Both phases were filtered by a 0.22 μm microporous membrane. The ratio of solvent and anti-solvent (*v*/*v*) was 1:10, 1:20, 1:40, 1:60. The other steps were identical to those described in Section 2.5.1, and all trials were completed in triplicate.

#### 2.5.3. Investigation of Sonication Time

The drug concentration of Bre was 100 mg/mL as the organic phase (solvent). Soybean lecithin was dissolved in ultrapure water to obtain a stabilizer solution with a concentration of 0.5% (*w*/*v*) as an aqueous phase (anti-solvent). Both phases were filtered by a 0.22 μm microporous membrane. The sonication power was set at 250 W, and the sonication times were set to 2, 5, 10, 15, and 20 min. The other steps were identical to those described in Section 2.5.1, and all trials were completed in triplicate.

### 2.6. BBD Optimization

Preliminary experiments showed that the drug concentration in DMSO and the anti-solvent-to-solvent ratio were important formulation variables that significantly affected the formation of a stable nanosuspension (Supplementary Materials Tables S1 and S2). Similarly, during the sonication process, the sonication time affected the reduction in particle size (Supplementary Materials Table S3). Therefore, the BBD was used to investigate the effects of different formulations and process variables on the formation and stability of the NS.

Bre-NS optimization was done using three factors at three levels in the BBD. The drug concentration in DMSO (A), the anti-solvent-to-solvent ratio (B), and the sonication time (C) were used as independent variables (Table 1). Their influences were determined by particle size (Y1), PDI (Y2), and the zeta potential (Y3). The experimental design was created using Design-Expert software (version 10.0.7, Stat-Ease Inc., Minneapolis, MN, USA), as shown in Table 2. The mathematical polynomial equation and the 3D surface plot were established, and the influence of formula variables on the response was analyzed.

### 2.7. Preparation of Bre-NS Freeze-Dried Powder

Bre-NS and mannitol were combined to provide an excellent protective effect during freeze-drying. To prepare Bre-NS freeze-dried powder, 7% mannitol was dissolved into Bre-NS; the mixture was pre-frozen at −80 °C for 3 h and sublimated at −40 °C for 1 h. It was heated to −15 °C at a constant speed for 14 h and kept at −15 °C for 2 h. It continued to be heated to 25 °C at a constant speed for 14 h. The final product was kept warm at 25 °C for 2 h out of the box.

### 2.8. Characterization of Bre-NS and Bre-NS Freeze-Dried Powder

#### 2.8.1. Scanning Electron Microscopy (SEM)

A scanning electron microscope (SU8010 Field Emission Scanning electron microscope, Hitachi, Chiyoda City, Japan) was used to determine the morphology of the Bre. An appropriate amount of Bre was evenly coated on the sample tank, the surface was sprayed with gold, and SEM observed the morphology.

#### 2.8.2. Transmission Electron Microscopy (TEM)

The samples were negatively stained with 2.0% (*w*/*v*) phosphotungstic acid, placed on a copper mesh coated with a carbon film, and dried at room temperature. The characterized surface morphology of Bre-NS and Bre-NS freeze-dried powder samples were examined using TEM (JEM 1200EX Transmission Electron Microscope, JEOL Co., Ltd., Tokyo, Japan).

#### 2.8.3. Powder X-ray Diffraction (PXRD)

X-ray diffraction of Bre, the soybean phospholipids, mannitol, their physical mixture, lyophilized Bre-NS (Bre-NS freeze-dried powder without mannitol) and the Bre-NS freeze-dried powder was performed using the XRD-6100 Lab XRD (Shimadzu, Kyoto, Japan). PXRD was performed in a symmetric reflection mode using Cu-Kα radiation generated at a tube pressure of 40 kV and a tube flow of 30 mA. The sample was placed on the flat aluminum sample rack for a scanning range of 3–60° at a scanning rate of 2°/min.

#### 2.8.4. Fourier Transform-Infrared Spectroscopy (FT-IR)

FT-IR of Bre, the soybean phospholipids, mannitol, their physical mixture, lyophilized Bre-NS, and the Bre-NS freeze-dried powder was performed using FT-IR (Nicolet IS50 Fourier transform-infrared spectrometer, Waltham, MA, USA). The samples were mixed with KBr and pressed at 10 MPa into prepared disks of 10 mm diameter. FT-IR was used to scan and record infrared spectra 32 times at a resolution of 2 cm^−1^ in the range of a 400–4000 cm^−1^ wave number.

#### 2.8.5. Differential Scanning Calorimetry (DSC)

The thermal properties of Bre, the soybean phospholipids, mannitol, their physical mixture, lyophilized Bre-NS, and the Bre-NS freeze-dried powder were evaluated using DSC (Q100, TA Co., Ltd., New Castle, DE, USA). The samples were placed in a sealed aluminum tray with a perforated cover. The measured temperature range was 10–300 °C, and the heating rate was 10 °C/min. Measurements were made under nitrogen flow at 50 mL/min.

### 2.9. Hemolysis and Coagulation

The hemolytic effect of Bre-NS on red blood cells (RBCs) was investigated by using fresh blood from Sprague-Dawley rats. In brief, blood samples were collected via cardiac puncture and immediately transferred to tubes. Fresh rat blood was centrifuged at 3000  rpm for 5 min to obtain red blood cells. RBCs were rinsed several times and diluted to 2% (*v*/*v*) with normal saline. RBC suspensions were mixed with normal saline (as a negative control) or mixed with deionized water (as a positive control) at a volume ratio of 1:1. The experimental design, shown in Table 2, utilized a series of concentrations of Bre-NS (2, 4 and 8 mg·mL^−1^) and a commercially available Bre injection (8 mg·mL^−1^) mixed with prepared 2% RBC suspensions (as tested groups) or deionized water (as blank controls). After mixing, these preparations were incubated at 37 ± 0.5 °C for 3 h to observe the hemolysis and coagulation reactions. The samples were centrifuged at 2500 rpm for 5 min. The absorbance of the supernatant was detected at 540 nm using an enzyme label, and the hemolysis rate (HR%) was calculated according to the following formula:

(1) $$\mathrm{Hemolysis}\;\mathrm{rate}\;\left(\%\right)=\frac{A_{t}-A_{n}}{A_{p}-A_{n}}\times 100\%,$$

in which A_t_, A_n_, and A_p_ are, respectively, the absorbance value of the test group, the negative control, and the positive control. All measurements were performed in triplicate.

### 2.10. Determination of Saturation Solubility

According to the method reported in the literature [28], the solubility of Bre, the physical mixture (equivalent to the prescription proportion of drugs, excipient mixture), and the Bre-NS freeze-dried powder in water were analyzed. In brief, the excess sample was added to 5 mL of distilled water and then oscillated in an oscillating gas bath at 100 rpm at 37 °C for 72 h. The final suspension was centrifuged at 12,000 rpm for 10 min to obtain the supernatant. It was filtered through a 0.22 μm microporous membrane to ensure that any undissolved Bre larger than 0.22 μm was separated from the dissolved drug. The concentration of Bre was determined by high-performance liquid chromatography (HPLC). HPLC was performed using an Agilent 1200 series system with ultraviolet detection and a ZORBAX SB-C18 (150 cm × 4.6 mm, 5 μm, Agilent, Santa Clara, CA, USA). The mobile phase consisted of 60% 0.01 mol/L phosphate and 40% methanol (HPLC grade) at a flow rate of 1.0 ml/min. Detection was at 335 nm.

### 2.11. In Vitro Release

Bre, the physical mixture, and the Bre-NS freeze-dried powder in water in the right amount, which contained the equivalent of 2 mg of Bre, were each placed into 200 mL of normal saline (pH = 6.70) in a dissolution cup with a rotation speed of 100 rpm and a temperature of 37 ± 0.5 °C. A 1 mL aliquot was withdrawn from samples at 5, 10, 15, 20, 30, 45, 60, and 120 min and was immediately replenished with 1 mL of released medium. Each sample was passed through a 0.22 μm microporous membrane, and the experiments were carried out in triplicate. HPLC determined the concentration of Bre, and the cumulative release rate was calculated at each time point. The determination method was the same as Section 2.10.

### 2.12. Stability Study

Although NS has various advantages, physical instability problems, such as particle aggregation, precipitation, and phase separation, may occur due to the active surface energy and Brownian motion. Therefore, the stability of an NS should be investigated during its storage period. The stabilities were investigated by storing the samples in a capillary vial at 4 °C and 25 °C. After storage for 0, 1, 3, 7, 15, and 30 days, the stability was evaluated by measuring particle size; all tests were performed in triplicate. The particle size, PDI, and zeta potential of Bre-NS were measured using a Malvern particle size analyzer. The experiments were carried out in triplicate.

### 2.13. Effects of Bre-NS on PC12 Cells

PC12 cells are rat adrenal pheochromocytoma cells that have the characteristics of nerve cells in physiological and biochemical aspects and are widely used to study cerebral ischemia, Alzheimer’s disease, and other diseases [37]. The safe dose range of Bre and Bre-NS was selected by evaluating the cytotoxicity on PC12 cells. PC12 cells were treated for 24 h with 200 μL of Bre, commercially available Bre injection, and Bre-NS with different concentrations (25, 50, 100, 200, and 400 μM) in each well. The viability of PC12 cells was determined using an MTT assay [38]; for this assay, 10 μL of 5 mg/mL of MTT solution was added to each well and incubated at 37 °C for 4 h. Then, the medium containing MTT was discarded, 100 μL of DMSO was added to dissolve the intracellular phagocyte, and the absorbancy was measured at 490 nm with a microplate analyzer.

(2) $$\mathrm{Cell}\;\mathrm{viability}\;\left(\%\right)=\frac{Abs_{sample}}{Abs_{control}}\times 100\%$$

### 2.14. Statistical Analysis

All values were expressed as the mean ± SD. The statistical analysis was made via a one-way analysis of variance using the SPSS Statistics 22.0 program. The differences were considered significant at *p*  <  0.05.

## 3. Results and Discussion

### 3.1. BBD Approach for Formulation Optimization

Bre-NS was developed using the anti-solvent precipitation method and was optimized with BBD software. As shown in Table 3, 17 experiments were generated according to the BBD. The results of actual and predicted values of the particle size (PS, Y1), PDI (Y2), and zeta potential (Y3) are shown in Table 4. According to the results of the 17 experiments, the particle size of Bre-NS ranged from 311.3 nm to 708.4 nm. The PDI changed from 0.175 to 0.524. The zeta potential ranged from −31 mV to −4.07 mV. The regression analysis is shown in Table 4. The coefficient of correlation (R^2^) indicated a great similarity between the actual experimental data and the predicted value. By analyzing the correlation coefficient R, adjustment coefficient R^2^, and prediction coefficient R^2^ among variables, it was observed that the quadratic model had a large R^2^ of 0.9883, 0.875, and 0.9403 in sequence. Therefore, the PS, PDI, and zeta potential of Bre-NS could be predicted by the quadratic equation of the response.

The regression equations derived by the model were as follows:

Y1 = 375.8 − 24.76A + 3.66B − 132.33C − 72.08AB + 56.5AC − 37.3BC + 48.51A^2^ + 45.86B^2^ + 51.89^2^ (3)

Y2 = 0.23 + 21.46A − 0.017B − 0.043C − 0.12AB − 0.036AC − 7.08BC + 0.06A^2^ + 0.065B^2^ + 15.03C^2^ (4)

Y3 = −27.84 + 5.42A − 6.08B − 0.96C − 5.51AB − 1.38AC + 0.9BC + 0.64A^2^ + 4.61B^2^ + 1.58C^2^ (5)

The variance analysis of the regression model for the particle size, PDI, and zeta potential was checked using *p*-values and the F test. The *p*-value was highly significant (*p* < 0.0001) and the lack-of-fit *p*-values were larger than 0.05 (0.1673, 0.6943, and 0.7078, respectively). These results showed that the fitted regression equation was good, explaining and predicting the results.

For the PS analysis, the drug concentration in DMSO (A) and the sonication time (C) were the two variables of all independent variables that showed a negative effect. Conversely, the anti-solvent-to-solvent ratio (B) displayed a positive effect on PS. The prepared Bre-NS was identified in the range of 311.3 nm to 708.4 nm. For the PDI, the drug concentration in DMSO (A) exhibited a positive effect, and the solvent ratio (B) and sonication time (C) showed a negative effect. The PDI value assured the homogeneity and size distribution of the NP [39]. For the zeta potential, the drug concentration in DMSO (A) exhibited a positive effect, and the solvent ratio (B) and sonication time (C) showed a negative effect.

**Figure 1:**
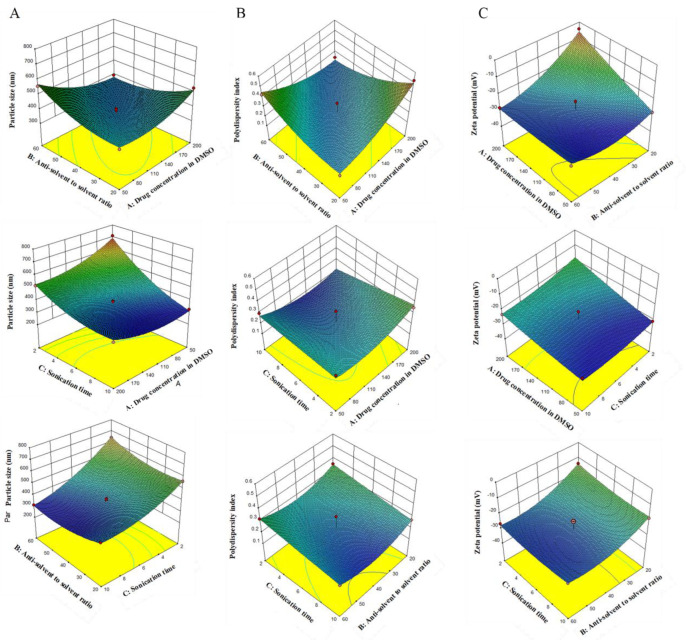
Effect of drug concentration in DMSO, anti-solvent to solvent ratio and sonication time on particle size (**A**), PDI (**B**), and zeta potential (**C**).

Response surface analysis was performed on the interaction between various factors, and the effects were plotted. On the response surface curve diagram, a steeper response surface represented a greater impact on the encapsulation efficiency. The effect of drug loading was also greater with a steeper curve. As illustrated in the 3D surface plot in Figure 1A,B, the sonication time had the steepest curve surface and the greatest influence on the particle size and PDI. Figure 1C shows that the inverse solvent-to-solvent volume ratio had the greatest influence on the zeta potential and the steepest curve surface.

The optimal preparation process identified by response surface optimization was described as follows: a certain amount of Bre was dissolved in DMSO to obtain a drug concentration of 170 mg/mL in solution. In addition, soybean lecithin was dissolved in ultra-pure water to obtain a stabilizer solution with a concentration of 0.5% (*w*/*v*). Both phases were filtered by a 0.22 μm microporous membrane. The sonication power was set to 250 W, and the probe was submerged midway within the liquid level. During an ice water bath ultrasound, the drug solution was rapidly injected into a stabilizer solution that was precooled to 4 °C; the ratio of solvent to anti-solvent was 1:60 (*v*/*v*), and the sonication time was performed for 9 min as continuous bursts of 3 s each with 1 s intervals. Then, the sample was immediately transferred to a centrifuge tube and centrifuged at 18 °C and 12,000 rpm for 10 min; the supernatant containing DMSO was removed, and then an equal amount of ultra-pure water was added. The centrifugation was repeated twice to obtain Bre-NS. The best-predicted values were a particle size of 311 nm, a PDI of 0.175, and potential of −28.14 mV. The actual values of the optimal formulation process were a particle size of 303.7 ± 7.3 nm, a PDI of 0.178 ± 0.015, and a potential of −31.10 ± 0.26 mV; these findings were consistent with the predicted values.

### 3.2. Characterization of Bre-NS and the Freeze-Dried Powder

#### 3.2.1. Characterization of Bre-NS and Its Freeze-Dried Powder

**Figure 2:**
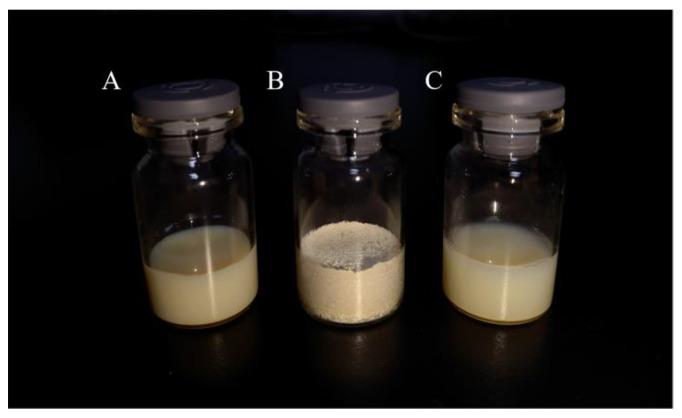
The physical appearance of Bre-NS (**A**), Bre-NS freeze-dried powder (**B**) and Bre-NS freeze-dried powder after redispersion (**C**).

Product appearance is one of the important objectives of the lyophilization process design and optimization [40]. As shown in Figure 2A, Bre-NS was translucent with blue opalescence, consistent with the reported description of an NS appearance [39]. As shown in Figure 2B,C, the freeze-dried Bre-NS powder had a plump appearance, a smooth surface, a uniform color, and a uniform structure with blue opalescence after redissolution. In addition, there was no obvious change in appearance between Bre-NS before freeze-drying and after redispersion. Therefore, it can be inferred that Bre-NS could remain stable after freeze-drying.

**Figure 3:**
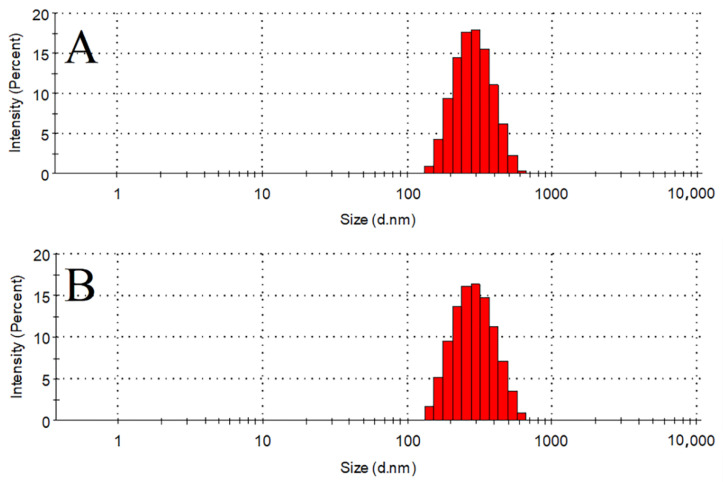
Particle size distribution of Bre-NS (**A**) and Bre-NS freeze-dried powder after redispersion (**B**).

As the BBD results showed, the particle size of the prepared Bre-NS was 303.7 ± 7.3 nm, and the PDI was 0.178 ± 0.015. The particle size of the redissolved Bre-NS was 302.2 ± 5.0 nm, and the PDI was 0.244 ± 0.022. As shown in Figure 3, there was no significant difference in particle size before versus after lyophilization.

#### 3.2.2. Morphology

**Figure 4:**
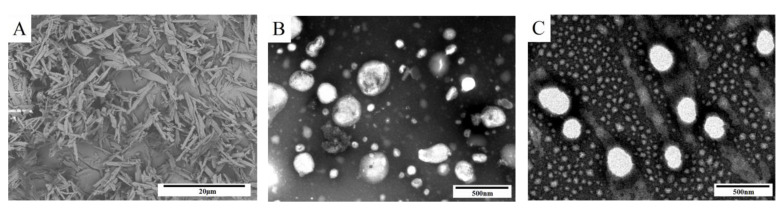
Microscopic observation.The SEM of Bre (**A**). Note: scale bar = 20 μm. The TEM of Bre-NS (**B**) and Bre-NS freeze-dried powder (**C**). Note: scale bar = 500 nm.

As can be seen from Figure 4A, Bre has a rod-like structure. The TEM micrograph of Bre-NS and the Bre-NS freeze-dried powder is shown in Figure 4B,C. The TEM micrographs revealed that Bre-NS and the Bre-NS freeze-dried powder were spherical in shape and had diameters of approximately 300 nm. The particles of all NSs showed a round shape and uniform size without any change.

#### 3.2.3. PXRD

**Figure 5:**
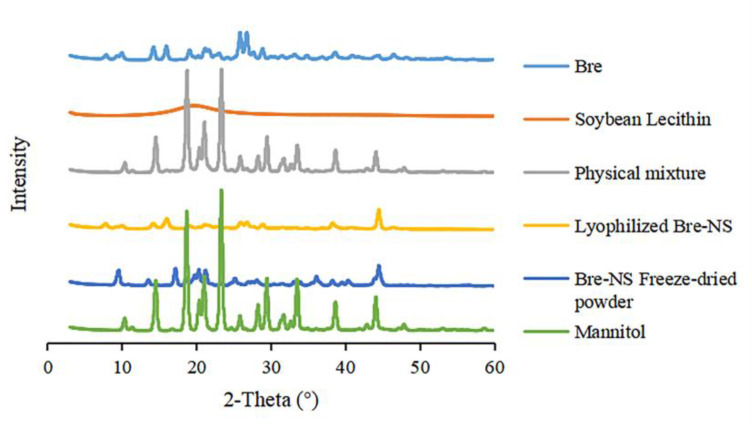
Powder X-ray diffraction (PXRD) pattern of Bre, soybean lecithin, physical mixture, lyophilized Bre-NS, the Bre-NS freeze-dried powder and mannitol.

Crystallinity is an important factor that affects physical and chemical properties, such as drug stability, solubility, and dissolution rate [41]. To confirm the crystalline state of Bre in Bre-NS, a PXRD analysis was conducted. PXRD of Bre, the excipients, mannitol, the physical mixture of Bre, lyophilized Bre-NS and the Bre-NS freeze-dried powder are shown in Figure 5. Crystalline peaks were obvious between 10° and 30°, and strong crystalline peaks occurred at the diffraction angles of 25.9° and 26.8°. The physical mixture also had weak crystalline peaks in this range, indicating that Bre had a specific crystal structure. The characteristic crystallization peaks of lyophilized Bre-NS and the Bre-NS freeze-dried powder still exist, indicating that the crystallization state has not changed. 

#### 3.2.4. FT-IR

**Figure 6:**
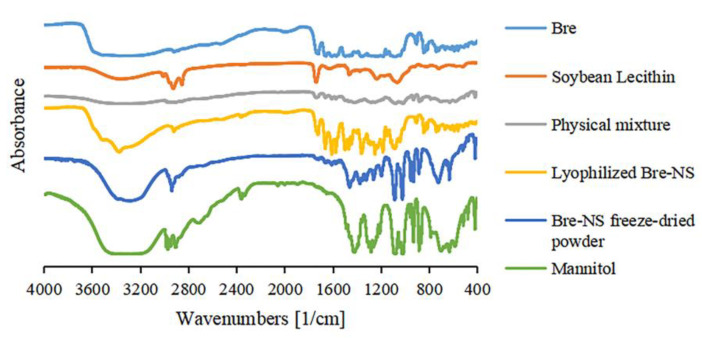
Fourier transform-infrared (FT-IR) spectrum of Bre, soybean lecithin, physical mixture, lyophilized Bre-NS, the Bre-NS freeze-dried powder and mannitol.

The FT-IR spectra of Bre, the soybean lecithin, the physical mixture, the lyophilized Bre-NS, the Bre-NS freeze-dried powder, and mannitol are shown in Figure 6. The characteristic absorption bands of Bre at 3373.87 cm^−1^, 2919.69 cm^−1^, 1149.88 cm^−1^, 1721.68 cm^−1^, 1248.24 cm^−1^, and 1660.46 cm^−1^ were still present in the physical mixture, lyophilized Bre-NS, and the Bre-NS freeze-dried powder, indicating that the chemical structure of the Bre-NS freeze-dried powder did not change after preparation. 

#### 3.2.5. DSC

**Figure 7:**
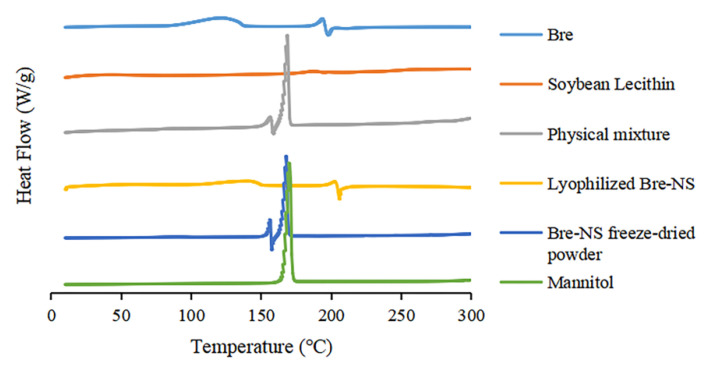
Differential scanning calorimetry (DSC) thermograms of Bre, soybean lecithin, the physical mixture, lyophilized Bre-NS, the Bre-NS freeze-dried powder and mannitol.

The DSC thermograms are depicted in Figure 7. The endothermic melting peak of Bre shifted to 197.79 °C, and the exothermic melting peak shifted to 193.75 °C; mannitol exhibited a characteristic exothermic melting peak at 167.95 °C. The exothermic peak and endothermic peak of lyophilized Bre-NS appear at the same position for Bre, indicating that the crystal form of the drug has not changed during the preparation process. The characteristic peaks of Bre and mannitol appeared in the DSC images of Bre-NS freeze-dried powder and the physical mixture with mannitol, but the characteristic peaks of Bre were shifted approximately 40 °C to the left. The decrease in the melting point of Bre could be attributed to the presence of mannitol and may be caused by the interaction between mannitol and Bre when they melt [42]. FT-IF, PXRD and DSC showed that the modification of the stabilizers and the preparation process had no significant effect on the crystal form and chemical structure of Bre.

### 3.3. Hemolysis and Coagulation

**Figure 8:**
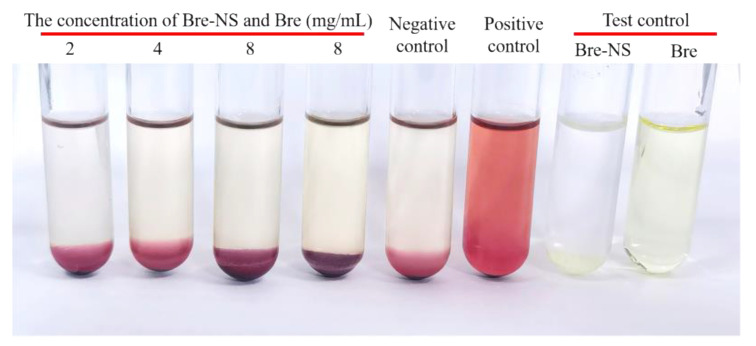
Hemolysis test results of the Bre-NS and commercially available Bre injection at different dilution ratios.

Bre-NS can be used as an intravenous injection because the drug should not be conducive to hemolysis. In this study, the hemolytic properties of Bre-NS were evaluated in vitro by observing whether adverse reactions, such as erythrocyte agglutination or hemolysis, occurred [43]. As shown in Figure 8, all RBCs in test tubes 1–4 sank, and the supernatant was colorless and clear. Reddish-brown flocculent precipitates were noted in the solutions of test tubes 1–5; these could be dispersed by gently inverting 3 times. These findings indicated that Bre-NS and commercially available Bre injection cause no hemolysis or RBC agglutination. The absorbance of the supernatant in each test tube was measured by enzyme conjugation. When the concentration of Bre-NS was 2, 4, or 8 mg/mL, the hemolysis rates were 1.17 ± 0.39%, 2.47 ± 0.81%, and 2.21 ± 0.90%, respectively. When the concentration of commercially available Bre injection was 8 mg/mL, the hemolysis rate was 3.51 ± 0.38%. Thus, the hemolysis rate of Bre-NS was lesser than 5%, indicating that Bre-NS had a non-hemolytic reaction.

### 3.4. In Vitro Release Studies

**Figure 9:**
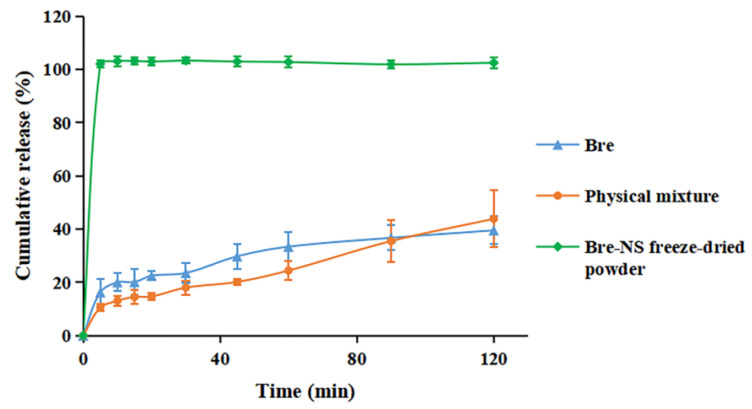
In vitro release curves of Bre, the physical mixture and Bre-NS freeze-dried powder.

As shown in Figure 9, the release of the Bre-NS freeze-dried powder reached 100% at 5 min, which was significantly higher than the drug substance and physical mixture. This was mainly due to the decrease in particle size and the increase in specific surface area on the nanometer scale [44]. However, the release of the physical mixture and Bre was very slow; only 43.88% and 39.55% were released at 2 h. This finding indicated that the NS lyophilized powder preparation of Bre had significantly increased dissolution in vitro.

### 3.5. Saturation Solubility

The saturation solubilities of Bre and the Bre-NS freeze-dried powder in distilled water were 0.0150 ± 0.0004 mg/mL and 0.0758 ± 0.0020 mg/mL, respectively. The solubility of Bre-NS was 5.07 times larger than that of Bre. The reduction in nanoparticle size significantly expanded the contact area with the solvent. The good stabilizer allowed the drug particles to be evenly dispersed in the water, thus preventing the agglomeration of particles and improving the stability of the drug [45].

### 3.6. Stability Study

**Figure 10:**
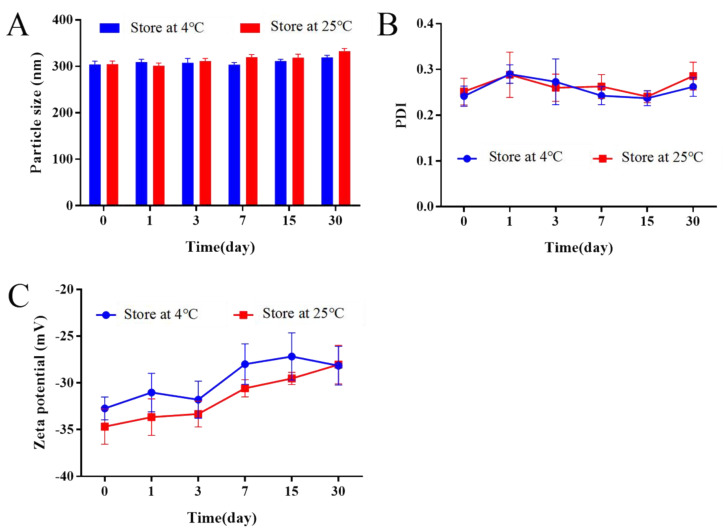
Changes in the particle size (**A**), PDI (**B**) and zeta potential (**C**) of Bre-NS stored at 4 °C or 25 °C and for a different duration (mean ± SD, *n* = 3).

Figure 10 showed that the particle size, PDI, and zeta potential of Bre-NS changed little and nonsignificantly (*p* > 0.05) when stored at 4 °C and 25 °C for 1 month. At 25 °C, the particle size increased from 304.8 ± 6.59 nm to 332.9 ± 5.62 nm; the PDI increased from 0.252 ± 0.029 to 0.286 ± 0.030, and the zeta potential decreased from −34.68 ± 1.88 mV to −28.04 ± 2.07 mV. At 4 °C, the particle size increased from 304.1 ± 7.05 nm to 319.4 ± 4.34 nm, the PDI increased from 0.242 ± 0.022 to 0.262 ± 0.021, and the zeta potential decreased from −32.72 ± 1.21 mV to −28.16 ± 2.07 mV. Amphiphilic soybean lecithin improved the wettability of nanoparticles, thus ensuring the good stability of drug particles. Soybean lecithin acted as a stabilizer of NS by forming an interfacial phospholipid membrane around a single drug particle; thus, the membrane could provide enough steric hindrance to prevent the aggregation of nanoparticles. 

### 3.7. Effects of Bre-NS on PC12 Cells

**Figure 11:**
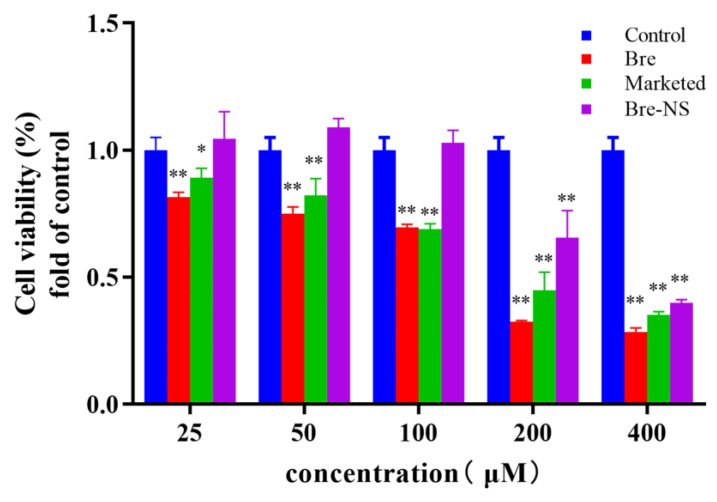
Cell viability of PC12 cells treated with different concentrations of Bre, Bre injection and Bre-NS for 24 h (*n* = 3) (compared with the control group, ** *p* < 0.001, * *p* < 0.05 compared with the Bre).

To investigate the safety of Bre-NS, PC12 cells were treated with different concentrations of Bre, a commercially available Bre injection, and Bre-NS. As shown in Figure 11, the cell viability decreased as the Bre concentration increased (especially when the concentration of Bre in the medium was 200 μM) to less than 50% after 24 h of culture, which suggests that Bre had a certain cytotoxicity and inhibited cell proliferation. In the concentration range of 25–200 μM, the cell viability of Bre-NS was significantly higher than that of a bulk drug (*p* < 0.001) and the commercial preparation (*p* < 0.05). At 200 μM, the relative viability of cells in the NS group was still higher than 50%, indicating that the NS formulation had little effect on PC12 cells and suggesting that its safety was higher than the bulk drugs or the Bre injection.

## 4. Conclusions

In this paper, Bre-NS was prepared by an ultrasound-assisted AP method, and the formulation process was optimized by BBD. The optimized formulation process should be set at the drug concentration of 170 mg/mL, an anti-solvent-to-solvent ratio of 1:60 (*v*/*v*), and a sonication time of 9 min. The actual values of the optimal formulation process were a particle size of 303.7 ± 7.3 nm, a PDI of 0.178 ± 0.015, and a potential of −31.10 ± 0.26 mV; these values were consistent with the predicted values. DSC, PXRD, and FT-IR results suggested that the crystal form and chemical structure did not change during the freeze-drying process. The hemolysis rates were all <5% without obvious hemolysis. The release rate of the Bre-NS freeze-dried powder reached 100% after 5 min, indicating that Bre-NS can significantly increase the dissolution rate of Bre in vitro. The solubility of Bre-NS was 5.07 times higher than that of Bre, which could effectively prevent the agglomeration of particles. After Bre-NS was stored at 4 °C and 25 °C for 1 month, the particle size, PDI, and zeta potential did not change significantly, indicating that Bre-NS could improve the stability of the drug. When the concentration of Bre was 200 µM, the relative viability of cells in the Bre-NS group was still higher than 50%, indicating that Bre-NS was safe. Bre-NS displayed good stability, increased solubility, and better in vitro release compared with Bre. Therefore, the results of this study can be a reference for the design of a delivery system of insoluble active components and effective parts in traditional Chinese medicine.

## Figures and Tables

**Table 1:** Box–Behnken design used to optimize the preparation process Bre-NS.

Variables	Levels		
	−1	0	1
Independent	-	-	-
A: Drug concentration in DMSO (mg/mL)	50	125	200
B: Anti-solvent-to-solvent ratio	20	40	60
C: Sonication time (min)	2	6	10
Dependent	Constraints		
Y1: Particle size (PS; nm)	Minimize		
Y2: Polydispersity index (PDI)	Minimize		
Y3: Zeta potential (mV)	Maximum ^1^		

^1^ Represents the maximum value of the absolute value of the zeta potential.

**Table 2:** Hemolysis experiment design.

Number	1	2	3	4	5	6	7	8
2% RBC Suspension/mL	2.5	2.5	2.5	2.5	2.5	-	-	-
0.9% NaCl/mL	2.2	2.2	2.2	2.2	2.5	2.5	4.7	4.7
Water/mL	-	-	-	-	-	-	-	-
Test solution/mL	0.3	0.3	0.3	0.3 ^1^	-	2.5	0.3	0.3 ^1^
Test solution/mg·mL^−1^	2	4	8	8	-	-	-	-

^1^ is a commercially available Bre injection.

**Table 3:** Effects of drug concentration in DMSO (A), anti-solvent-to-solvent ratio (B), and sonication time (C) on particle size (Y1), polydispersity index and (Y2), and zeta potential (Y3) of Bre-NS during preparation.

Run	Independent Variables			Dependent Variables		
	A (mg/mL)	B	C (min)	Y1 (nm)	Y2	Y3 (mV)
1	125	40	6	386.8	0.192	−22.8
2	125	60	10	311.3	0.227	−27.9
3	125	40	6	392.5	0.175	−28.4
4	200	40	10	357.0	0.194	−23.0
5	200	40	2	517.7	0.349	−19.5
6	125	40	6	361.0	0.318	−28.5
7	125	20	10	380.2	0.267	−18.5
8	50	40	10	321.7	0.297	−29.0
9	125	60	2	641.5	0.319	−26.6
10	50	20	6	404.3	0.211	−28.0
11	200	60	6	391.9	0.245	−28.2
12	200	20	6	527.1	0.524	−4.1
13	50	60	6	557.4	0.425	−30.1
14	50	40	2	708.4	0.307	−31.0
15	125	40	6	376.9	0.218	−30.9
16	125	20	2	561.2	0.353	−13.6
17	125	40	6	361.8	0.225	−28.6

**Table 4:** Output data of the Box–Behnken design analysis of Bre-NS.

Source df		Y1: Particle Size				Y2: Polydispersity Index				Y3: Zeta Potential			
		Sum of Squares	Mean Square	F Value	*p*-Value	Sum of Squares	Mean Square	F Value	*p*-Value	Sum of Squares	Mean Square	F Value	*p*-Value
Model	9	2.18 × 10^5^	24,203.95	65.85	<0.0001	0.12	0.013	5.45	0.018	777.45	86.38	12.25	0.0016
A	1	4905.45	4905.45	13.35	0.0081 *	6.48 × 10^−4^	6.48 × 10^−4^	0.27	0.6216	234.69	234.69	33.28	0.0007 *
B	1	107.31	107.31	0.29	0.6057	2.42 × 10^−3^	2.42 × 10^−3^	0.99	0.3521	295.61	295.61	41.92	0.0003 *
C	1	1.40 × 10^5^	1.40 × 10^5^	381.11	<0.0001 *	0.015	0.015	6.05	0.0435 *	7.41	7.41	1.05	0.3394
AB	1	20,779.22	20,779.22	56.53	0.0001 *	0.061	0.061	24.99	0.0016 *	121.33	121.33	17.21	0.0043 *
AC	1	12,769	12,769	34.74	0.0006 *	5.26 × 10^−3^	5.26 × 10^−3^	2.16	0.185	7.56	7.56	1.07	0.3348
BC	1	5565.16	5565.16	15.14	0.006 *	9.00 × 10^−6^	9.00 × 10^−6^	3.70 × 10^−3^	0.9532	3.24	3.24	0.46	0.5196
A2	1	9909.32	9909.32	26.96	0.0013 *	0.015	0.015	6.33	0.0401 *	1.7	1.7	0.24	0.638
B2	1	8856.29	8856.29	24.1	0.0017 *	0.018	0.018	7.36	0.0301 *	89.53	89.53	12.7	0.0092 *
C2	1	11,336.05	11,336.05	30.84	0.0009 *	2.06 × 10^−6^	2.06 × 10^−6^	8.49 × 10^−4^	0.9776	10.49	10.49	1.49	0.262
Residual	7	2572.89	367.56	-	-	0.017	2.43 × 10^−3^	-	-	49.36	7.05	-	-
Lack of Fit	3	1756.75	585.58	2.87	0.1673	4.74 × 10^−3^	1.58 × 10^−3^	0.51	0.6943	13.27	4.42	0.49	0.7078
Pure Error	4	816.14	204.03	-	-	0.012	3.07 × 10^−3^	-	-	36.09	9.02	-	-
Cor Total	16	220,400	-	-	-	0.14		-	-	826.81		-	-
-	-	R^2^ = 0.9883	R^2^_Adj._ = 0.9733	R^2^_Pred._ = 0.9733		R^2^ = 0.875	R^2^_Adj._ = 0.7143	R^2^_Pred._ = 0.3027		R^2^ = 0.9403	R^2^_Adj._ = 0.8635	R^2^_Pred._ = 0.675	

* is Significant values at *p* < 0.05.

## Data Availability

The authors declare that all data supporting the findings are available within the paper or are available from the authors upon request.

## Supplementary Materials

The following supporting information can be downloaded at: https://www.mdpi.com/article/10.3390/pharmaceutics14050923/s1. Table S1: Particle size, PDI and zeta potential for different drug concentrations in DMSO; Table S2: Particle size, PDI and zeta potential for the different solvent-to-anti-solvent volume ratios; Table S3: Particle size, PDI and zeta potential for different sonication times.
