# Supplementary material for: Preparation, Characterization, and Evaluation of Breviscapine Nanosuspension and Its Freeze-Dried Powder
Source: Pharmaceutics. 2022 Apr 24;14(5):923. doi: 10.3390/pharmaceutics14050923 (PMC9143020; doi:10.3390/pharmaceutics14050923)
Supplement: Supplementary file 1 [file pharmaceutics-14-00923-s001.zip › pharmaceutics-1628898-supplementary.pdf]

pharmaceutics

Supplementary Materials: Preparation, Characterization and Evaluation of Breviscapine Nanosuspension and Its Freeze-Dried Powder

Ting Zhang, Xixi Li, Juewen Xu, Jingbao Shao, Meihong Ding, Senlin Shi *

Tabel S1. Effects of different drug concentrations in DMSO on different drug concentrations in DMSO (mean ± SD, n=3).

**Drug concentration in DMSO (mg/mL)**	**Particle size (nm)**	**PDI**	**Zeta potential (mV)**
50	329.17±12.82	0.203±0.011	-25.53±3.13
100	387.60±1.40	0.274±0.014	-24.87±1.50
200	436.37±10.25	0.338±0.037	-16.57±1.70
300	470.50±1.25	0.411±0.013	-7.80±0.95
400	470.50±5.91	0.378±0.082	-2.93±1.06

Tabel S2. Effects of different solvent and antisolvent volume ratios on particle size, PDI and Zeta potential（mean±SD, n=3）.

Solvent to anti-solvent volume ratio	Particle size (nm)	PDI	Zeta potential (mV)
1：10	575.80±28.66	0.438±0.053	-2.95±0.57
1：20	482.00±6.42	0.430±0.035	-20.20±1.14
1：40	393.20±5.88	0.278±0.012	-27.37±0.47
1：60	354.90±5.00	0.273±0.029	-28.63±3.30

Tabel S3. Particle size, PDI and Zeta potential for different sonication time (mean±SD, n=3).

Sonication time (min)	Particle size (nm)	PDI	Zeta potential (mV)
2	404.20±3.70	0.213±0.015	-27.80±0.26
5	299.67±6.73	0.204±0.014	-29.10±1.73
10	261.93±3.41	0.172±0.010	-27.30±1.25
15	255.37±9.25	0.165±0.022	-26.70±0.44
20	245.90±3.82	0.162±0.001	-27.50±2.55
